# Simple Puncture in Empyema

**Published:** 1888-04

**Authors:** 


SIMPLE PUNCTURE IN EMPYEMA.—WÖLFLER, of Gratz, recently reported to the Society of Physicians, of Styria, a case of empyema cured by simple puncture. The empyema had developed spontaneously, and was probably due to tuberculosis. Puncture was made with a trocar between the sixth and seventh ribs, on the left side, and the pus evacuated by siphon drainage. The lower end of the rubber tube communicated with a bottle filled with antiseptic fluid, and was left in till no more pus escaped. Healing took place very rapidly, and this method seemed to be preferable to extensive resection of ribs. Wölfler has recently treated three patients successfully by this method. They were told to carry the bottle, with the drainage apparatus, till no more pus escaped.

